# Rationale and Design of the “PRECISION-CT” Study—A Prospective Evaluation of Coronary CTA Integration for Strategy Improvement and Optimization of PCI in Chronic Coronary Syndrome

**DOI:** 10.3390/diagnostics16050715

**Published:** 2026-02-27

**Authors:** Dimitrios V. Moysidis, Nicolai V. Bogert, Sorin Giusca, Ronny R. Buechel, Andreas A. Giannopoulos, Grigorios Korosoglou

**Affiliations:** 1 Department of Nuclear Medicine, Cardiac Imaging, University Hospital and University of Zurich, 8006 Zurich, Switzerlandandreas.giannopoulos@usz.ch (A.A.G.); 2 Department of Cardiology, Vascular Medicine and Pneumology, GRN Hospital Weinheim, 69469 Weinheim, Germany; 3 Cardiac Imaging Center Weinheim, Hector Foundation, 69469 Weinheim, Germany

**Keywords:** chronic coronary syndromes, percutaneous coronary intervention (PCI), coronary computer tomography angiography (CCTA), CT-guided PCI

## Abstract

**Background:** Coronary computed tomography angiography (CCTA) is a well-established key diagnostic modality for ruling out obstructive coronary artery disease (CAD) in patients with suspected chronic coronary syndromes (CCSs) and low to intermediate pre-test probability. The widespread availability of preprocedural CCTA data in CCS patients undergoing percutaneous coronary intervention (PCI), however, creates a new opportunity for image-guided procedural planning. **Objective:** The PRECISION-CT study (Prospective Evaluation of Coronary CTA Integration for Strategy Improvement and Optimization of Non-Emergent PCI) aims to evaluate the impact of CCTA-guided PCI on patient safety and clinical outcomes. **Methods:** PRECISION-CT is a prospective, two-center, randomized controlled trial, enrolling patients with CCS scheduled for elective PCI due to obstructive CAD by CCTA. Patients are randomized 1:1 to either CCTA-guided PCI or standard angiography-guided PCI. In patients randomized to CCTA-guided PCI, advanced post-processing of CCTA datasets provides specific procedural planning recommendations based on the detailed assessment of coronary artery takeoff, lesion location and plaque characteristics. In addition, real-time integration of the advanced CCTA post-processing is available in the catheterization laboratory during the PCI procedure. Patients randomized to angiography-guided PCI are treated according to routine clinical practice. **Results**: The primary endpoint is a composite procedural safety and efficacy score including: (i) need for intravascular imaging, (ii) procedural complications, (iii) post-procedural high-sensitivity troponin T elevation, (iv) contrast media, (v) radiation exposure, and (vi) length of hospital stay. Secondary endpoints include major cardiac adverse events such as cardiac death, non-fatal myocardial infarction, target-lesion reintervention and probable or definitive stent thrombosis during 1 year of follow-up. **Conclusions:** The PRECISION-CT study will provide pragmatic evidence on the ability of CT-guided PCI in patients with CCS to optimize procedural outcomes. These findings may help inform the broader adoption of image-guided precision revascularization strategies in interventional cardiology.

## 1. Introduction

Coronary computed tomography angiography (CCTA) has evolved to become a cornerstone of non-invasive cardiac imaging for patients with suspected chronic coronary syndromes (CCSs), based on current recommendations [1]. Its widespread adoption, particularly in the initial evaluation of patients, helps to rule out obstructive coronary artery disease (CAD) and to avoid unnecessary invasive coronary angiography (ICA) procedures in most patients with low to intermediate pre-test probability [2]. On the other hand, in patients with obstructive CAD, including those with high-grade (>90%) lesions or with intermediate lesions of functional significance, who are subsequently referred for ICA and percutaneous coronary intervention (PCI), CCTA datasets could be used to support pre- and periprocedural PCI planning [3].

This evolution presents a unique opportunity to enhance procedures by integrating anatomical and functional information derived from CCTA into the cardiac catheterization laboratory. Thus, beyond its diagnostic capabilities, CCTA offers a comprehensive assessment of coronary anatomy, including coronary artery takeoff, plaque morphology, lesion length, vessel size, and overall disease distribution [4,5,6,7]. These features are crucial in determining PCI complexity and for informing strategic decisions such as vascular access, stent selection and sizing, and the need for intravascular imaging or additional periprocedural lesion preparation techniques [4]. While randomized controlled trials such as the Precise Procedural and PCI Plan (P4) study are currently investigating the clinical equivalence of CT-guided PCI versus intravascular-imaging-guided PCI [4,8], real-world data on the practical implementation, feasibility, and impact of CCTA-guided PCI in routine practice remain limited [9]. To address this gap, the PRECISION-CT study (Prospective Evaluation of Coronary CTA Integration for Strategy Improvement and Optimization of Non-Emergent PCI) has been designed.

**Figure 1:**
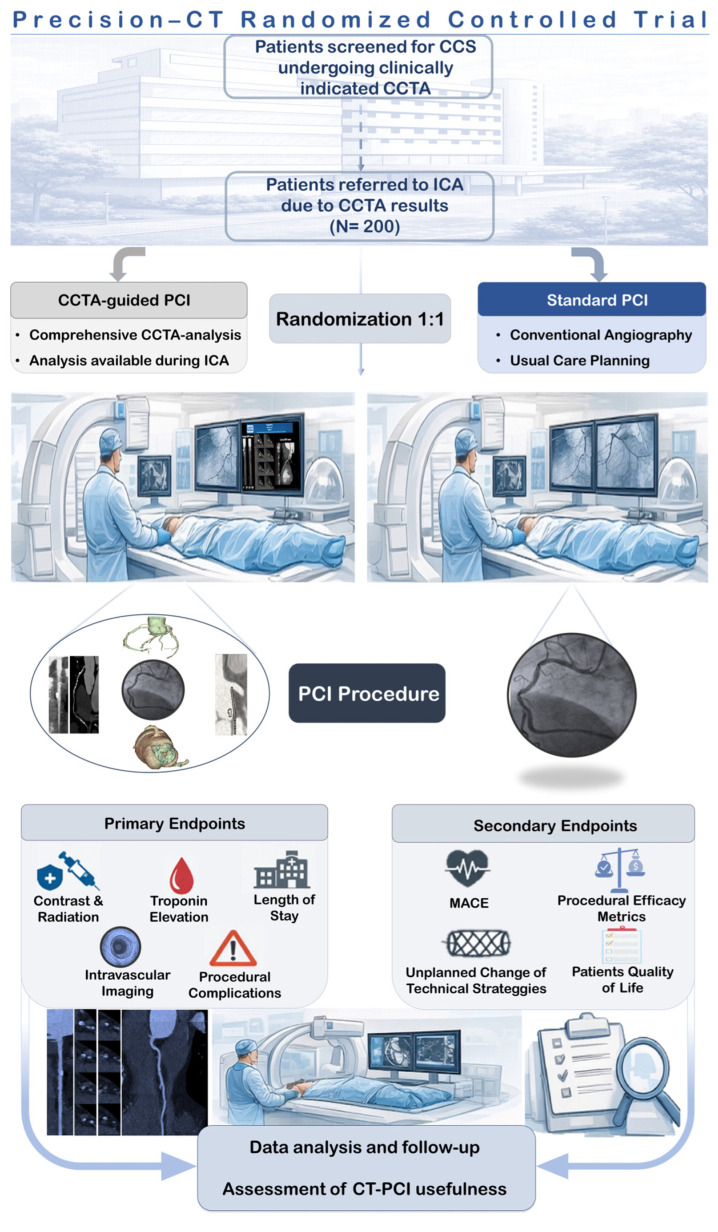
Methodological aspects and flowchart of the trial.

The aim of PRECISION-CT is to determine whether CCTA-guided PCI planning improves procedural strategy and execution in patients undergoing non-emergent revascularization. By integrating pre-PCI CCTA findings with procedural characteristics and clinical outcomes, the trial aims to clarify how CT-based planning can refine interventional strategies, reduce procedural uncertainty and reduce the need for intravascular imaging, thus optimizing resource utilization, procedural time, costs, patient safety, contrast volume, and radiation exposure. All these aspects may thereby ultimately introduce a more personalized and evidence-based approach to PCI. Figure 1 summarizes the methodological aspects and flowchart of the trial. 

Importantly, the PRECISION-CT study evaluates the incremental value of structured, quantitative, and real-time CCTA integration into the catheterization laboratory compared with standard-of-care CCTA-informed PCI. This design reflects contemporary clinical practice, where CCTA is frequently available prior to ICA, and focuses on whether enhanced integration of existing imaging data can improve procedural planning, efficiency, and safety.

## 2. Materials and Methods

### 2.1. Study Design and Setting

The PRECISION-CT study is an investigator-initiated, prospective, randomized, dual-center clinical trial designed to evaluate the impact of extended CCTA analysis on procedural planning and outcomes of PCI. The trial will be conducted at the GRN Hospital Weinheim, Germany, with image post-processing and advanced CCTA analyses performed at the University Hospital Zurich (USZ). Data transfer and analysis will be carried out according to Good Clinical Practice (GCP) standards and the Declaration of Helsinki. The study protocol has received approval from the local ethics committee (S-436/2025) and is registered in the German Registry of Clinical Studies (DRKS00038939). All participants will provide written informed consent prior to enrollment. Patient safety will be ensured via bail-out criteria, documentation of omitted measures, and monitoring of complications by the hospital’s steering ethics committee.

### 2.2. Patient Population

Eligible patients are adults (≥18 years) with suspected CCS who undergo clinically indicated CCTA and are subsequently referred for ICA based on CCTA findings. Recruitment will be performed consecutively among patients undergoing clinically indicated CCTA at the GRN Hospital Weinheim (approximately 700 annually). Patients will be informed about this study during the standard ICA consent process.

### 2.3. Inclusion Criteria

• Manifest obstructive CAD confirmed on CCTA with functionally significant coronary stenosis >50% or anatomically significant coronary stenosis >90% in one or more coronary vessels and appropriate anatomy for PCI.
• Signed informed consent.

### 2.4. Exclusion Criteria

• Emergent PCI (e.g., acute coronary syndromes); therefore, CT processing does not delay urgent or emergent care.
• Poor image quality, non-diagnostic or incomplete CCTA datasets.
• No need for PCI despite positive CCTA (as per ICA or invasive physiology like RFR or FFR).
• Left-main disease with >50% diameter stenosis of the left main requiring treatment.
• Age < 18 years.
• Pregnancy.
• Lack of signed informed consent.

### 2.5. CCTA Acquisition and Analysis

Coronary computed tomography angiography will be performed in accordance with Society of Cardiovascular Computed Tomography (SCCT) guidelines. Prior to image acquisition, intravenous beta-blockade (metoprolol) will be administered in individually titrated doses to achieve a target heart rate below 65 beats per minute whenever feasible. Sublingual nitrates will be routinely administered for coronary vasodilation.

The acquisition protocol is individualized based on patient-specific parameters, including heart rate, heart rhythm, and body habitus. In the majority of cases, ECG-triggered prospective axial acquisition will be employed. Patients with persistently elevated heart rates (>80 bpm) despite beta-blocker administration or with frequent ectopic beats will undergo ECG-triggered prospective arrhythmia-adapted axial scanning or spiral acquisition with retrospective ECG-gated reconstruction. In patients with stable heart rates below 65 bpm and a body mass index (BMI) < 28 kg/m^2^, a prospectively ECG-triggered high-pitch spiral acquisition protocol will be performed [10].

All examinations will be performed using a third-generation dual-source CT scanner (SOMATOM Force, Siemens Healthineers, Forchheim, Germany) capable of 384-slice acquisition (2 × 192 detector rows). Scan parameters will be adapted to patient BMI and included a tube voltage range of 80–120 kVp and tube current of 290–560 mA. Images will be reconstructed using a 512 × 512 matrix, with a slice thickness of 0.8 mm and an increment of 0.4 mm. For contrast enhancement, 50–60 mL of iodinated contrast medium (iopromide, Ultravist^®^, Bayer Healthcare, Berlin, Germany) will be administered via an antecubital vein at a flow rate of 5 mL/s, followed by a 50 mL saline flush at the same injection rate. Image acquisition will be timed to the arterial contrast phase using ECG gating.

CCTA scans will be analyzed by experienced readers with >5 years of CCTA experience, level 2/3 certified by the German Society of Cardiology. CAD-RADS 2.0 will be used to categorize the degree of lumen narrowing as follows: (0) none (0%), (1) minimal (1–24%), (2) mild (25–49% stenosis), (3) moderate (50–69% stenosis), (4) severe (70–99% stenosis), or (5) chronic total occlusions (100%) [11].

### 2.6. Randomization and Study Arms

Randomization will be performed after completion of CCTA analysis and prior to invasive coronary angiography. Participants will be randomized (1:1) into two groups using sequentially numbered, opaque, sealed envelopes. The randomization sequence will be generated by an independent study nurse. Sequentially numbered, opaque, sealed envelopes will be prepared centrally and stored securely. After written informed consent and patient inclusion, the envelope will be opened immediately before PCI by a study nurse not involved in outcome assessment. Operator blinding is not feasible due to the nature of our study. For patients allocated to the intervention arm, anonymized CT datasets will be transferred securely to the image-analysis site in Switzerland (Nuclear Medicine Department) using the USZ Secure Transfer Platform (https://transfer.usz.ch/). There, detailed CT analysis and three-dimensional reconstructions will be performed using CE-marked post-processing software (GE Healthcare Advantage Workstation, AW, Version 4.7). CT data transfer requires approximately 10 min, and structured image analysis and procedural planning require approximately 30 min, ensuring cost- and time-effectiveness. Only elective PCI cases will be included, which undergo ambulatory CCTA and are then scheduled for elective PCI based on CCTA and clinical data, or if required additional stress test preprocedural findings; therefore, CT processing will not delay urgent or emergent care. The analysis results will be returned to the German study site prior to PCI and made available during the procedure. Treating operators will be informed of group allocation immediately before the PCI procedure to minimize allocation-related scheduling or case-selection bias. Participating operators have >5 years of experience in interventional cardiology and are board-certified with a formal focus in Interventional Cardiology of the German Cardiac Society (DGK).

**Intervention arm**: Full CCTA analysis, including 3D reconstructions, is displayed on an integrated monitor (3D Tabcard, CPQ-1511578) in the catheterization laboratory during PCI and can directly guide procedural decisions.

**Control arm**: The operator reviews the standard clinical CCTA report and images prior to ICA/PCI according to institutional standard operating procedures, without structured quantitative post-processing, procedural planning recommendations, or real-time visualization in the catheterization laboratory.

### 2.7. PCI Procedure and CT-Guided Planning

Interventional cardiologists will receive a standardized CCTA report prior to the PCI procedure during the pilot study phase for all patients and during the randomized study for the patients randomized to the CCTA-guided PCI arm. The report contains annotated images and textual descriptions covering optimal angiographic projections, reference vessel diameters, lesion length, calcium distribution, and bifurcation anatomy to guide stent and device selection. A representative file report is provided in the Supplementary Materials, illustrating how quantitative and actionable information is communicated consistently to the operator. Procedural planning includes:

• Maximal Intensity Projection (MIP) images of the coronary tree.
• Exact location and height of coronary ostia.
• Axial images of coronary arteries.
• Multiplanar reformation (MPR) images of coronary arteries.
• Short-axis cross-sectional images.
• Lesion length.
• Bifurcation anatomy and vessel tortuosity, as well as a suggested provisional stenting versus a two-stent approach.
• Calcification burden, including the arc of the calcification in degrees and the calcification length.
• Lesion morphology, including the presence and extent of mixed and non-calcified plaques.
• Optimal C-arm angulation angles.
• The need for lesion preparation (rotational atherectomy or intravascular lithotripsy), choice of guiding catheter, stenting strategy, and projection angles may be adapted accordingly.

Intravascular imaging will not be discouraged in either study arm and will be encouraged in both arms in certain clinical scenarios and based on current guideline recommendations. Thus, operators may perform IVI based on pre-specified bail-out criteria, including uncertain lesion length or morphology, suboptimal stent expansion or malapposition, complex bifurcation anatomy, heavy calcification, or any safety concern. All IVI use, including specific indications for imaging, will be prospectively documented. Notably, the “need for IVI” in the composite endpoint reflects procedural escalation and resource utilization and not an adverse clinical event.

### 2.8. Data Collection

Data will be collected prospectively and will be entered into a standardized database. The collected variables include:

▪ **Demographics**: age, gender, date of admission, and length of hospitalization.
▪ **Indications for CCTA and ICA**: typical angina pectoris, atypical angina pectoris, non-anginal chest pain, or presence of dyspnea.
▪ **Medical history and cardiovascular risk factors**: prior myocardial infarction, hypertension, dyslipidemia, diabetes mellitus, current or prior cigarette smoking, chronic obstructive pulmonary disease, chronic kidney disease, atrial fibrillation, or peripheral artery disease.
▪ **Cardiac medications at baseline.**
▪ **Echocardiographic data**: left ventricular ejection fraction (LVEF) or presence of regional wall motion abnormalities.
▪ **CCTA data:**
  o Anatomic lesion location, including segment number and classification into proximal, mid, or distal vessel.
  o Ostial involvement and measurement of coronary ostium height from the aortic annulus.
  o Takeoff angle of right and left coronary ostia, assessed using clock-face reference.
  o Plaque composition (calcified, non-calcified or mixed based on the presence of the calcified content by visual criteria of >80%, 20–80% and <20% calcium content) [12].
  o Presence of high-risk plaque features, including: positive remodeling, napkin-ring sign, low attenuation plaque (<30 HU) or spotty calcification [13].
  o Total coronary calcium score quantified using the Agatston score.
▪ **Invasive procedural data**: Comprehensive data from invasive coronary procedures will be collected, including: use of advanced intracoronary imaging (IVUS if necessary), use of physiological assessment (FFR or iFR/RFR if necessary), number of stents implanted, their total length, stent diameters, the use of re-dilation, including the use of scoring balloons and the corresponding pressures, and the use of lesion modification techniques (rotablation or/and intravascular lithotripsy), and bifurcation stenting strategies including provisional versus bifurcation stenting if necessary.
▪ **Procedural outcomes and metrics**: Final TIMI flow grade, residual stenosis in percentage, procedural success, defined as final TIMI 3 flow with <20% residual stenosis in the treated segment and absence of in-hospital major adverse procedural events, additional procedural metrics included total fluoroscopy time (minutes), radiation exposure (mGy), contrast volume used (ml), and total procedure duration (minutes).
▪ **Operator feedback and satisfaction:**
  o Use of CT-based projections during PCI.
  o Prediction of stent dimensions (length and diameter) based on CCTA measurements.
  o Prediction of landing zones for stent deployment.
  o Satisfaction with CCTA planning (1–5 scale for both primary and secondary operators).
  o Perceived benefit of CCTA-derived data on ostial angle, lesion length, plaque quality and projection guidance, helping with reducing procedure time, fluoroscopy exposure and complications.
  o Whether CCTA planning helped in avoiding unnecessary PCI or reducing the number of stents required for lesion treatment.

### 2.9. Study Endpoints

Primary composite endpoint (score 0–6):

The primary composite endpoint was designed to capture procedural efficiency and safety rather than long-term clinical outcomes. Individual components were selected to reflect aspects of PCI that may be directly influenced by improved preprocedural anatomical understanding and planning, including resource utilization, procedural complexity, and periprocedural myocardial injury. The composite score is intended as a pragmatic measure of procedural optimization rather than a surrogate for clinical outcome.

1. Need for intravascular imaging (IVUS).
2. Occurrence of major procedural complications (perforation, severe dissection, stent malapposition, or under-expansion requiring post-dilatation, main/side branch occlusion).
3. Post-procedural high-sensitivity troponin T elevation >5-fold compared to baseline at 24 h.
4. Contrast volume > 200 mL as established in prior interventional studies.
5. Radiation dose > predefined threshold (estimated effective dose of >16.1 mSv) according to institutional and published reference levels for PCI procedures [14].

Hospital stay > 2 days. Secondary endpoints (exploratory):

In-hospital MACE and complications or the need for repeated cardiac catheterization with or without reintervention during the index hospitalization or within 30 days after the procedure.

Long-term major adverse cardiac events (MACE): cardiac death, probable or definitive stent thrombosis, non-fatal myocardial infarction, or target-lesion revascularization at 12 and 24 months.

Guiding catheter changes or unplanned use of lesion preparation techniques.

Procedural efficiency metrics (procedure duration, fluoroscopy time, material consumption, and cost-effectiveness).

Patient-reported outcomes: quality of life using the SAQ-7 questionnaire.

Clinical events and complications are defined according to established consensus criteria, including Academic Research Consortium (ARC) definitions for stent thrombosis and target-lesion failure and Society for Cardiovascular Angiography and Interventions (SCAI) criteria for procedural complications. Imaging-derived findings are reported separately and are not classified as clinical events unless they meet these standardized definitions.

Figure 1 summarizes the methodological aspects and flowchart of the trial.

### 2.10. Initial Observational Feasibility Phase

**Figure 2:**
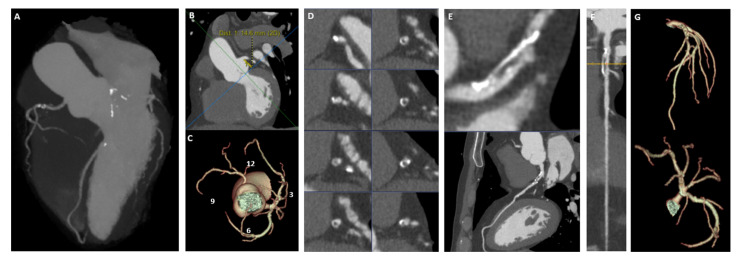
Overview of the CCTA image data provided to PCI operators in the pilot phase. (**A**) Initial assessment using Maximum Intensity Projection (MIP) images and MIP-based representation of coronary anatomy. (**B**) Assessment of coronary ostial engagement, including the height of the coronary artery takeoff from the aortic annulus (**B**) and the clock-based location of the ostia around the aortic circumference (**C**). (**D**) Cross-sectional views of the coronary vessels, with specific reference to affected vessels. (**E**) Curved Multiplanar Reconstruction (MPR) images to evaluate vessel stenosis and the degree of narrowing along the coronary arteries. (**F**) Straight MPR images to assess coronary anatomy, including the length and location of plaques within the vessels. (**G**) Suggested angiographic projection angles for optimized visualization of stenoses during PCI, to guide accurate intervention.

Prior to the randomized trial, an initial observational feasibility phase will be conducted, including approximately 60 consecutive patients who undergo clinically indicated CCTA followed by subsequent PCI procedures in the GRN Hospital Weinheim. In this phase, anonymized CCTA datasets will be analyzed at the University Hospital Zurich to evaluate image quality, completeness of reconstructions, and clinical applicability of the CCTA-derived procedural planning parameters. The purpose of this phase is to assess workflow integration, verify technical feasibility and post-processing, and refine the standardized reporting template for use in the randomized study. Data obtained during this phase will not be used for hypothesis testing but will serve to validate study logistics and optimize the design of the subsequent randomized trial. In particular, the use of intravascular ultrasound (IVUS) is encouraged during this pilot study phase to verify CCTA findings using intravascular imaging. Figure 2 presents the basic information provided by the Cardiac Imaging Department of the University Hospital of Zurich for each patient during the pilot phase. This information includes maximum MIP images and MIP-based representations of the coronary anatomy; assessment of coronary ostial takeoff (clock-face orientation and height from the aortic annulus); curved and straight MPR images; orthogonal cross-sectional views of the coronary arteries; recommended optimal angiographic projection angles; and procedural suggestions for PCI planning.

### 2.11. Statistical Analysis

Descriptive statistics will be used to characterize the patient population, procedural details, and outcomes. Categorical variables will be reported as counts and percentages and compared using the chi-square or Fisher’s exact test. Continuous variables will be assessed for normality using the Shapiro–Wilk test. Variables that are normally distributed will be presented as mean ± standard deviation and compared using *t*-tests, while non-normally distributed variables will be presented as median (interquartile range) and compared using Mann–Whitney U tests. To assess the impact of CT planning on procedural outcomes, univariate and multivariable logistic regression models will be applied. Variables with a *p*-value < 0.10 in univariate analysis will be included in the multivariable models using a backward stepwise approach. Odds ratios (OR) with 95% confidence intervals (CI) will be reported. The primary analysis will be performed according to the intention-to-treat principle.

Procedural metrics (e.g., contrast volume, fluoroscopy time, radiation dose, procedure duration) will additionally be assessed using linear regression models. Receiver operating characteristic (ROC) curve analyses will be performed to evaluate the diagnostic performance of specific CT features (e.g., ostial angle, lesion length, plaque morphology) for predicting procedural complications or strategy changes. The area under the curve (AUC) with 95% CI will be calculated. Interobserver and intraobserver variability for CCTA-derived measurements (e.g., ostial height, takeoff angle, lesion length) will be assessed using intraclass correlation coefficients (ICC) for continuous variables and Cohen’s κ for categorical variables.

The planned sample size of *n* = 200 patients (100 per arm) who will ultimately undergo PCI is based on prior published data on CT-guided PCI and institutional experience [3]. Since the components of the primary composite procedural optimization score have not previously been combined in a randomized trial, exact event rates are unknown. For sample size calculation, we assumed an event rate of approximately **50%** and a **20% absolute reduction** in the CCTA-guided arm, based on extrapolation from observational CT-guided PCI studies and institutional experience. While these assumptions may not be completely fulfilled in our real-world study setting, they provide a reasonable framework to ensure >80% statistical power to detect differences in the primary composite endpoint with a two-sided α level of 0.05 and type II error (β) of 0.20. Randomization will be performed using sequentially numbered, opaque, sealed envelopes opened immediately before PCI. Strict handling procedures are in place, and all envelope use is logged to maintain allocation concealment and integrity. Exploratory post hoc analyses will assess whether the effect of CCTA-guided planning differs according to age, sex, lesion complexity, calcification severity, bifurcation involvement, and ostial lesion location. Missing data will be handled using multiple imputation if >5% of values are missing for key variables; otherwise, complete case analysis will be performed. All statistical tests will be two-sided, with *p*-values < 0.05 considered statistically significant. Analyses will be performed using R (v4.3.1) and SPSS (v29).

## 3. Discussion

The integration of CCTA into PCI planning represents an important evolution from conventional “ad hoc” approaches toward a more comprehensive and patient-centered procedural strategy. CCTA-guided PCI involves the comprehensive assessment of lesion morphology, plaque characteristics, vessel dimensions, and coronary anatomy using high-resolution imaging prior to the catheterization procedure. This process provides interventional cardiologists with critical insights to anticipate lesion complexity and to plan device strategy accordingly, often without the need for additional intravascular imaging when high-quality diagnostic CCTA is available, and lesion characteristics are well defined [1,15]. Notably, CCTA-guided PCI has already been reported to provide distinct advantages in anatomically complex cases such as chronic total occlusions (CTOs), prior coronary artery bypass grafting (CABG), anomalous coronary origins, or equivocal ICA [16,17,18].

The value of CCTA-guided PCI is increasingly recognized for both clinical outcomes and operational efficiency. Preprocedural planning with CCTA enables more informed patient selection and resource allocation. Delineating the extent and distribution of CAD enhances procedural preparedness, minimizes intraprocedural uncertainty, especially for less experienced operators, and facilitates informed, shared decision-making between clinicians and patients [19,20]. In particular, CCTA provides a detailed anatomical roadmap that may indicate the need for surgical revascularization in cases of severe multivessel or diffuse disease and support more tailored procedural strategies, including stent sizing, landing zone prediction, and projection angles. These capabilities support a transition toward more proactive and structured interventional planning, with evidence suggesting potential reductions in procedure time, radiation exposure, and contrast volume when using CT-based planning [21].

Despite promising results, several barriers remain to the widespread implementation of CCTA-guided PCI. Access to high-end CT technology, skilled personnel for image interpretation, and integrated visualization software may still vary across institutions and healthcare systems [1,15,22,23]. In addition, limitations in scan quality due to arrhythmia, severe obesity, or kidney dysfunction may preclude its use in selected populations [16,17,24]. Furthermore, the adoption of CCTA-guided PCI depends on a collaborative infrastructure that supports integration between imaging specialists and interventionalists. Ongoing trials such as P4 (NCT05253677), CT-COMPASS (NCT06280638), and CT-CTOPCI (NCT04549896) will be instrumental in validating the impact of this strategy on long-term outcomes, procedural efficiency, and clinical decision-making. Our study will complement these efforts by providing comprehensive, real-world evidence from a contemporary cohort undergoing both CCTA and invasive coronary angiography, with emphasis on specific clinical, anatomical, and procedural factors that influence the decision to perform CCTA-guided PCI. By linking detailed clinical parameters, advanced CCTA-derived anatomical features, and procedural data, our work will offer novel insights into patient selection and outcome prediction.

A key design feature of PRECISION-CT is its pragmatic “all-comer” randomized approach. While the incremental value of CCTA-guided PCI is expected to be most pronounced in anatomically complex lesions, restricting enrollment only to high-risk subsets (e.g., severely calcified or high SYNTAX score cases) would have possibly introduced spectrum bias or prolonged recruitment and potentially overestimated the treatment effect. Our design enables robust subgroup analyses in patients with complex anatomy—where 3D coronary mapping and plaque characterization are most likely to influence strategy—while maintaining the generalizability and feasibility required for future real-world implementation. Furthermore, the study workflow is designed to reflect real-world practice, where operators typically review CCTA reports and/or imaging data within 24 h prior to or sometimes even immediately before PCI rather than several days or weeks in advance. Based on this 24 h preprocedural window, operators can mentally simulate the procedure, considering different device strategies, lesion preparation techniques, and angiographic projections. Notably, lesion preparation tools, such as specific atherectomy burr sizes, lithotripsy catheters and scoring balloons in different sizes and lengths and microcatheters or guide catheter shapes, will constantly be available in our institution. Real-time access to the CCTA dataset during PCI, on the other hand, is expected to enhance procedural flexibility, allowing adjustments to device selection and techniques based on the additional intraprocedural findings. This pragmatic approach maximizes the clinical applicability of CCTA-guided PCI while preserving workflow feasibility.

Adding to the current landscape of CCTA-guided PCI in clinical practice, our study will provide pragmatic data on the correlation between preprocedural CCTA findings and actual procedural conduct and outcomes. This includes evaluating the concordance between CCTA-derived lesion assessment and angiographic complexity [21,25], the utility of CCTA in patients with prior CABG or CTO [16], and the procedural consequences of specific CT-based findings such as calcification, lesion length, and bifurcation involvement. Future directions will include the development of integrated risk models that combine CCTA and echocardiographic data of cardiac magnetic resonance metrics to guide revascularization strategies, as well as the potential use of AI-based CCTA analysis and photon-counting CT to further improve image resolution, predictive accuracy, and operator independence [26]. Ultimately, our findings will help bridge the gap between CCTA-derived anatomical assessment and real-world interventional decision-making, providing a foundation for broader implementation of structured, image-guided PCI planning in routine clinical practice.

Several limitations should be acknowledged. First, operators cannot be blinded to group allocation, introducing potential performance bias. Second, this study is powered for procedural and efficiency endpoints rather than hard clinical outcomes, which will be assessed on an exploratory basis. Third, the control group reflects contemporary CCTA-informed practice rather than the absence of CCTA, which may attenuate observed differences but which enhances real-world applicability. Finally, results from two centers with advanced imaging expertise may not be directly generalizable to all clinical settings.

## 4. Conclusions

The PRECISION-CT study is designed to evaluate the incremental procedural value of structured, quantitative CCTA-guided planning for elective PCI in a contemporary real-world cohort. By focusing on procedural efficiency, safety, and resource utilization, this trial aims to identify clinical and anatomical scenarios in which enhanced CCTA integration may meaningfully improve PCI safety, planning and execution. PRECISION-CT will complement ongoing randomized trials and inform the future implementation of image-guided, personalized revascularization strategies.

## Data Availability

No new data were created or analyzed in this study. Data sharing is not applicable to this article.

## Supplementary Materials

The following supporting information can be downloaded at: https://www.mdpi.com/article/10.3390/diagnostics16050715/s1.

## References

[1] Vrints C., Andreotti F., Koskinas K.C., Rossello X., Adamo M., Ainslie J., Banning A.P., Budaj A., Buechel R.R., Chiariello G.A. (2024). 2024 ESC Guidelines for the Management of Chronic Coronary Syndromes. Eur. Heart J..

[2] Korosoglou G., Thiele H., Silber S., Schmitz T., Tiefenbacher C., Landmesser U., Helfen A., Nowak B., Bernhardt P., Baldus S. (2023). Bedarfs- und leitliniengerechte Diagnostik bei symptomatischer obstruktiver koronarer Herzkrankheit mittels Kardio-CT und MRT. Kardiologie.

[3] Ochs M., Breitbart P., Sultan A., Hell M., Schulz-Menger J., Lurz P., Tillmanns C., Tesche C., Linke A., Achenbach S. (2025). DGK-Positionspapier zur Schnittbildgebung Teil I: Kardiale Computertomographie zur periprozeduralen Planung und Durchführung von kardialen Interventionen. Kardiologie.

[4] Sandoval Y., Leipsic J., Collet C., Ali Z.A., Azzalini L., Barbato E., Cavalcante J.L., Costa R.A., Garcia-Garcia H.M., Jones D.A. (2025). Coronary Computed Tomography Angiography to Guide Percutaneous Coronary Intervention: Expert Opinion from a SCAI/SCCT Roundtable. J. Cardiovasc. Comput. Tomogr..

[5] Serruys P.W., Kotoku N., Nørgaard B., Garg S., Nieman K., Dweck M., Bax J., Knuuti J., Narula J., Perera D. (2023). Computed Tomographic Angiography in Coronary Artery Disease. EuroIntervention.

[6] Shabbir A., Attia A., Weber N.M., Alhassan M., Radike M., Lane C.M., Challa A.B., Wamil M. (2025). The Emerging Role of Computed Tomography Coronary Angiography in the Left Main Stem Percutaneous Coronary Intervention. Rev. Cardiovasc. Med..

[7] Kolossváry M., Szilveszter B., Merkely B., Maurovich-Horvat P. (2017). Plaque Imaging with CT—A Comprehensive Review on Coronary CT Angiography Based Risk Assessment. Cardiovasc. Diagn. Ther..

[8] Douglas P.S., Nanna M.G., Kelsey M.D., Yow E., Mark D.B., Patel M.R., Rogers C., Udelson J.E., Fordyce C.B., Curzen N. (2023). Comparison of an Initial Risk-Based Testing Strategy vs Usual Testing in Stable Symptomatic Patients with Suspected Coronary Artery Disease: The PRECISE Randomized Clinical Trial. JAMA Cardiol..

[9] Stalikas N., Bouisset F., Mizukami T., Tajima A., Munhoz D., Ikeda K., Sonck J., Wyffels E., Wilgenhof A., Astudillo P. (2025). Clinical Utility of Coronary CT Angiography to Guide PCI: A Survey among P4 Investigators. Int. J. Cardiovasc. Imaging.

[10] Lee S., Giesen A., Mouselimis D., Weichsel L., Giannopoulos A.A., Nunninger M., Renker M., André F., Frey N., Korosoglou G. (2025). Composite Cardiac Computed Tomography Angiography Score for Improved Risk Assessment in Chronic Coronary Syndromes. Sci. Rep..

[11] Cury R.C., Leipsic J., Abbara S., Achenbach S., Berman D., Bittencourt M., Budoff M., Chinnaiyan K., Choi A.D., Ghoshhajra B. (2022). CAD-RADS^TM^ 2.0—2022 Coronary Artery Disease-Reporting and Data System: An Expert Consensus Document of the Society of Cardiovascular Computed Tomography (SCCT), the American College of Cardiology (ACC), the American College of Radiology (ACR), and the North America Society of Cardiovascular Imaging (NASCI). J. Cardiovasc. Comput. Tomogr..

[12] Gitsioudis G., Schüssler A., Nagy E., Maurovich-Horvat P., Buss S.J., Voss A., Hosch W., Hofmann N., Kauczor H.-U., Giannitsis E. (2015). Combined Assessment of High-Sensitivity Troponin T and Noninvasive Coronary Plaque Composition for the Prediction of Cardiac Outcomes. Radiology.

[13] Giusca S., Schütz M., Kronbach F., Wolf D., Nunninger P., Korosoglou G. (2021). Coronary Computer Tomography Angiography in 2021-Acquisition Protocols, Tips and Tricks and Heading beyond the Possible. Diagnostics.

[14] Stocker T.J., Abdel-Wahab M., Möllmann H., Deseive S., Massberg S., Hausleiter J. (2022). Trends and predictors of radiation exposure in percutaneous coronary intervention: The PROTECTION VIII study. EuroIntervention.

[15] Gulati M., Levy P.D., Mukherjee D., Amsterdam E., Bhatt D.L., Birtcher K.K., Blankstein R., Boyd J., Bullock-Palmer R.P., Writing Committee Members (2021). 2021 AHA/ACC/ASE/CHEST/SAEM/SCCT/SCMR Guideline for the Evaluation and Diagnosis of Chest Pain: A Report of the American College of Cardiology/American Heart Association Joint Committee on Clinical Practice Guidelines. J. Am. Coll. Cardiol..

[16] Hong S.-J., Kim B.-K., Cho I., Kim H.-Y., Rha S.-W., Lee S.-H., Park S.M., Kim Y.H., Chang H.-J., Ahn C.-M. (2021). Effect of Coronary CTA on Chronic Total Occlusion Percutaneous Coronary Intervention: A Randomized Trial. JACC Cardiovasc. Imaging.

[17] Jones D.A., Beirne A.-M., Kelham M., Rathod K.S., Andiapen M., Wynne L., Godec T., Forooghi N., Ramaseshan R., Moon J.C. (2023). Computed Tomography Cardiac Angiography Before Invasive Coronary Angiography in Patients with Previous Bypass Surgery: The BYPASS-CTCA Trial. Circulation.

[18] Kumar S., Opolski M.P., Ahn J.-M., Collet C., Carvalho P.E.P., Jaffer F., Werner G.S., Leipsic J., Kim B.-K., Cavalcante J. (2026). The Role of Coronary Computed Tomography Angiography in Chronic Total Occlusion Percutaneous Coronary Intervention. JACC Cardiovasc. Interv..

[19] Collet C., Onuma Y., Andreini D., Sonck J., Pompilio G., Mushtaq S., La Meir M., Miyazaki Y., de Mey J., Gaemperli O. (2018). Coronary Computed Tomography Angiography for Heart Team Decision-Making in Multivessel Coronary Artery Disease. Eur. Heart J..

[20] Chan Z., Carvalho P.E.P., Cavalcante J.L., Lesser J., Cheng V., Brilakis E.S., Sandoval Y. (2024). Left Main Coronary CT-Guided Percutaneous Coronary Intervention: Role of Virtual Planning and Wireless Physiology. JACC Cardiovasc. Interv..

[21] van den Buijs D.M.F., Poels E.M., Willems E., Cottens D., Dotremont K., De Leener K., Meekers E., Ferdinande B., Vrolix M., Dens J. (2025). Three-Dimensional CT for Preprocedural Planning of PCI for Ostial Right Coronary Artery Lesions: A Randomized Controlled Pilot Trial. Circ. Cardiovasc. Interv..

[22] Valle J.A., Tamez H., Abbott J.D., Moussa I.D., Messenger J.C., Waldo S.W., Kennedy K.F., Masoudi F.A., Yeh R.W. (2019). Contemporary Use and Trends in Unprotected Left Main Coronary Artery Percutaneous Coronary Intervention in the United States: An Analysis of the National Cardiovascular Data Registry Research to Practice Initiative. JAMA Cardiol..

[23] Grines C.L., Box L.C., Mamas M.A., Abbott J.D., Blankenship J.C., Carr J.G., Curzen N., Kent W.D.T., Khatib Y., Matteau A. (2023). SCAI Expert Consensus Statement on Percutaneous Coronary Intervention Without On-Site Surgical Backup. J. Soc. Cardiovasc. Angiogr. Interv..

[24] Andreini D., Belmonte M., Penicka M., Van Hoe L., Mileva N., Paolisso P., Nagumo S., Nørgaard B.L., Ko B., Otake H. (2024). Impact of Coronary CT Image Quality on the Accuracy of the FFRCT Planner. Eur. Radiol..

[25] Sonck J., Nagumo S., Norgaard B.L., Otake H., Ko B., Zhang J., Mizukami T., Maeng M., Andreini D., Takahashi Y. (2022). Clinical Validation of a Virtual Planner for Coronary Interventions Based on Coronary CT Angiography. JACC Cardiovasc. Imaging.

[26] Shiyovich A., Singh A., Blair C.V., Cardoso R., Huck D., Peng G., Shaw L.J., Leipsic J.A., Gräni C., Antoniades C. (2026). Photon-Counting Computed Tomography in Cardiac Imaging. JACC Cardiovasc. Imaging.

