# Supplementary material for: Rationale and Design of the “PRECISION-CT” Study—A Prospective Evaluation of Coronary CTA Integration for Strategy Improvement and Optimization of PCI in Chronic Coronary Syndrome
Source: Diagnostics (Basel). 2026 Feb 27;16(5):715. doi: 10.3390/diagnostics16050715 (PMC12984380; doi:10.3390/diagnostics16050715)
Supplement: Supplementary file 1 [file diagnostics-16-00715-s001.zip › Example Case.pptx]

## Slide 1
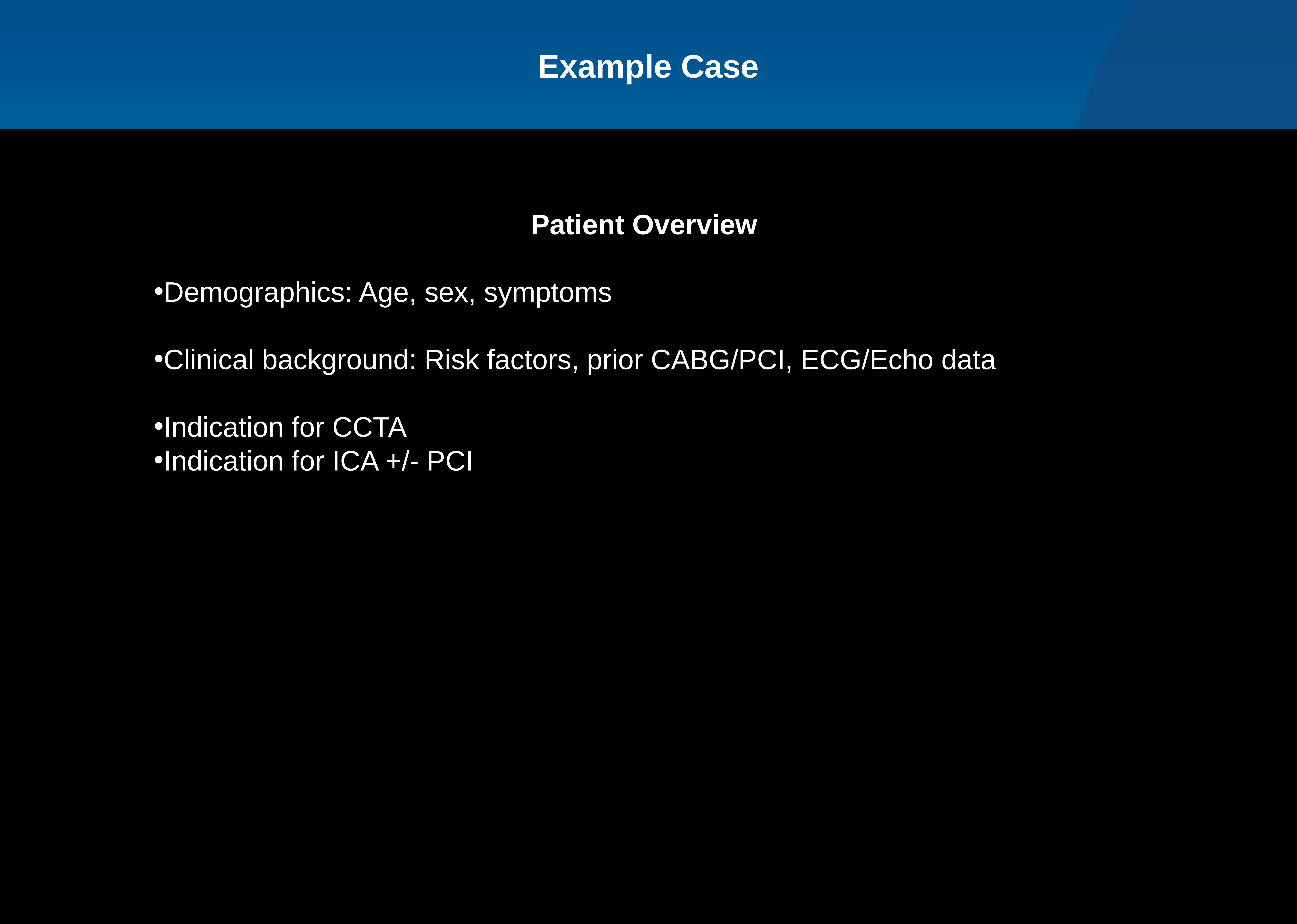

Example Case
Patient Overview
Demographics: Age, sex, symptoms
Clinical background: Risk factors, prior CABG/PCI, ECG/Echo data
Indication for CCTA
Indication for ICA +/- PCI

## Slide 2
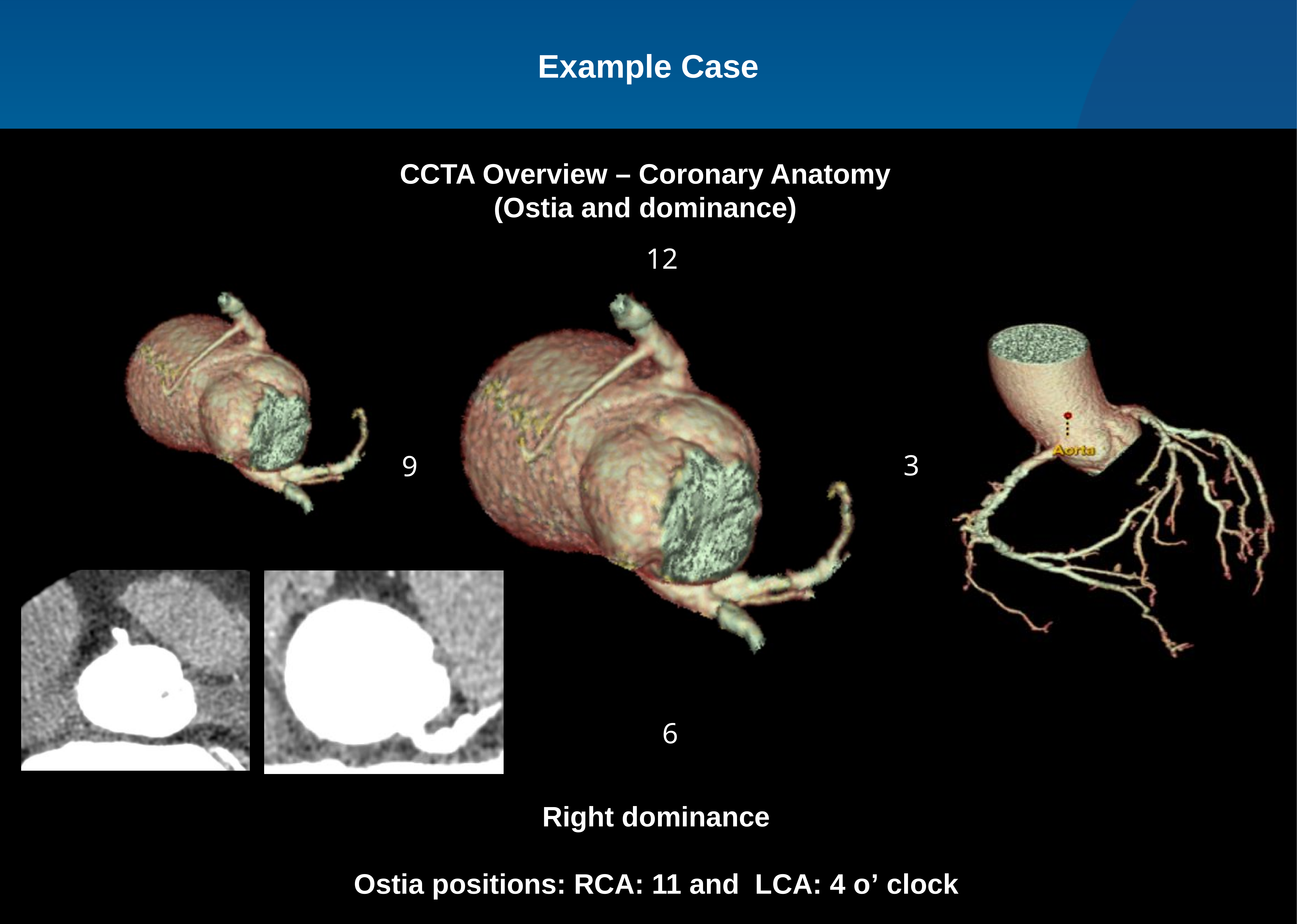

Example Case
CT-guided PCI
Case 1
CCTA Overview – Coronary Anatomy
(Ostia and dominance)
12
3
9
6
Right dominance
Ostia positions: RCA: 11 and LCA: 4 o’ clock

## Slide 3
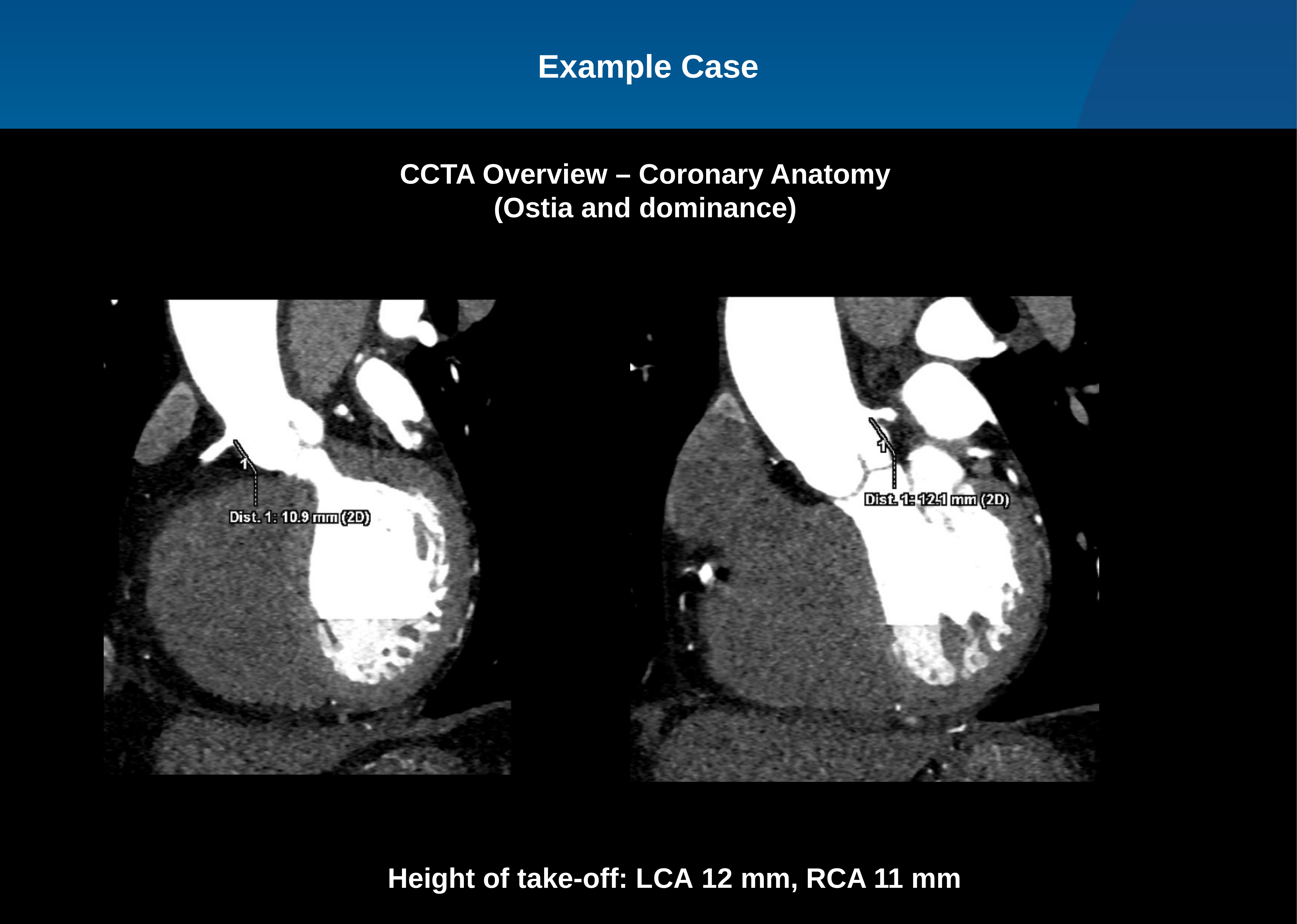

Example Case
CT-guided PCI
Case 1
CCTA Overview – Coronary Anatomy
(Ostia and dominance)
Height of take-off: LCA 12 mm, RCA 11 mm

## Slide 4
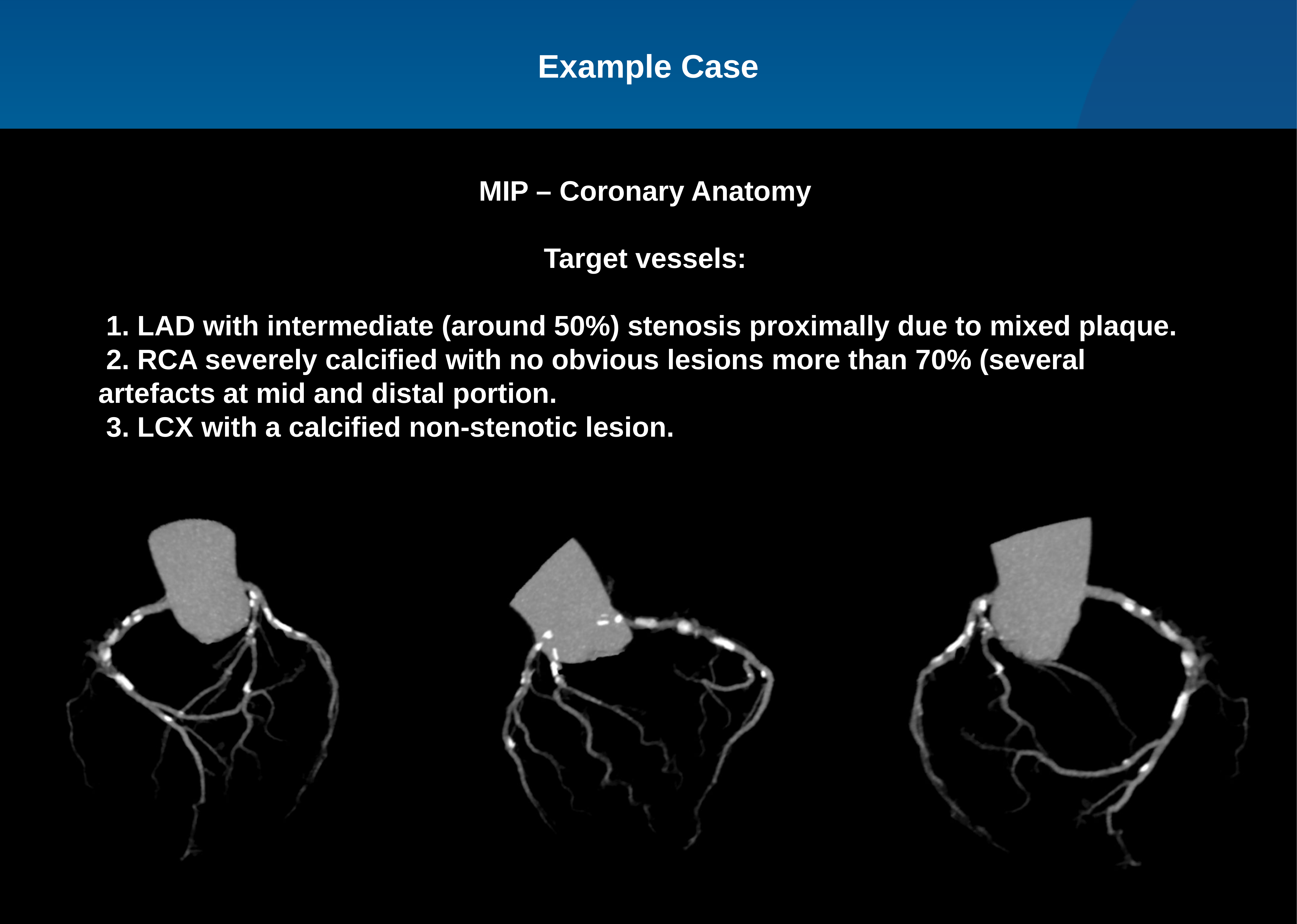

Example Case
CT-guided PCI
Case 1
MIP – Coronary Anatomy
Target vessels:
 1. LAD with intermediate (around 50%) stenosis proximally due to mixed plaque.
 2. RCA severely calcified with no obvious lesions more than 70% (several artefacts at mid and distal portion.
 3. LCX with a calcified non-stenotic lesion.

## Slide 5
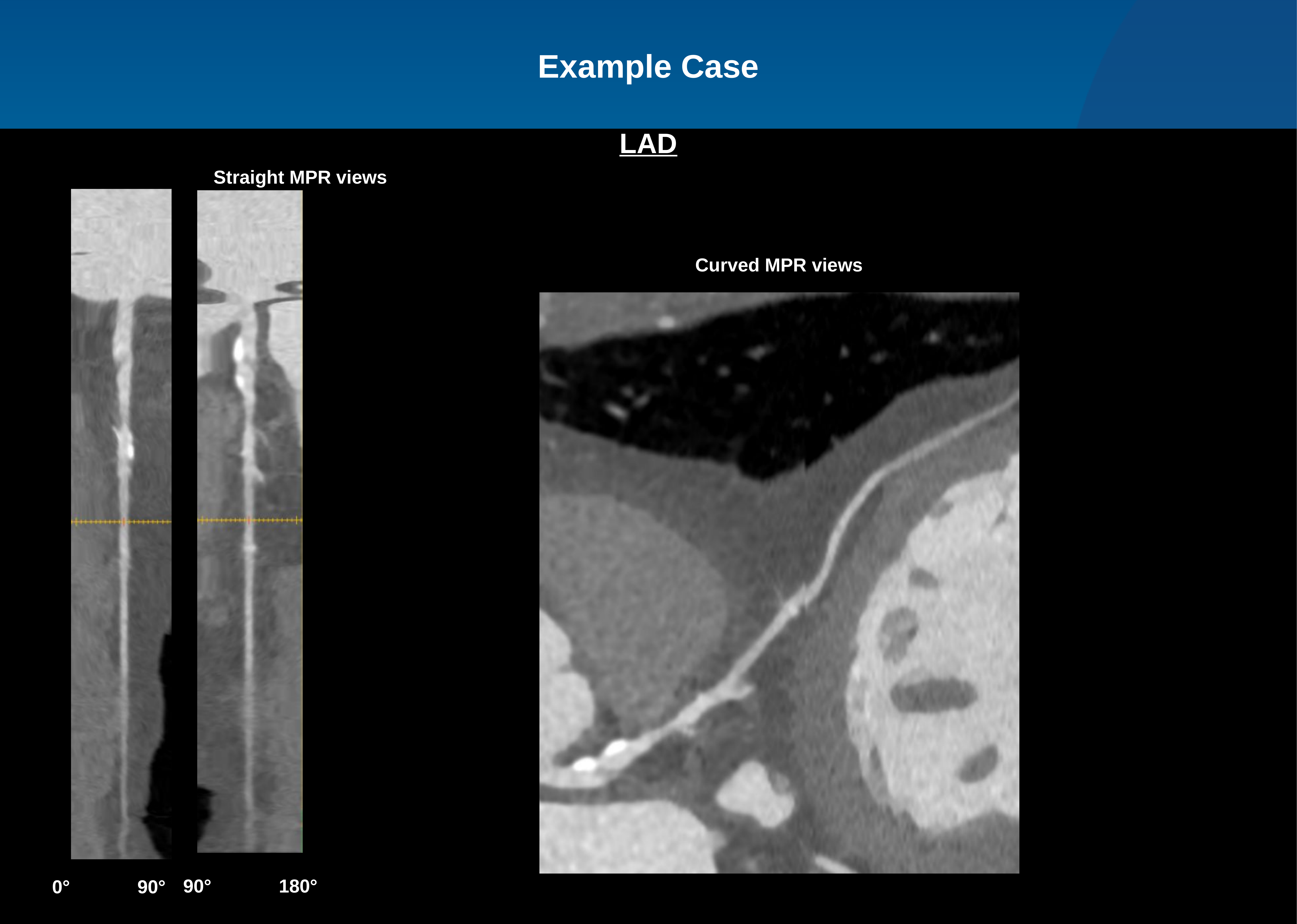

Example Case
CT-guided PCI
Case 1
LAD
Straight MPR views
Curved MPR views
90° 180°
0° 90°

## Slide 6
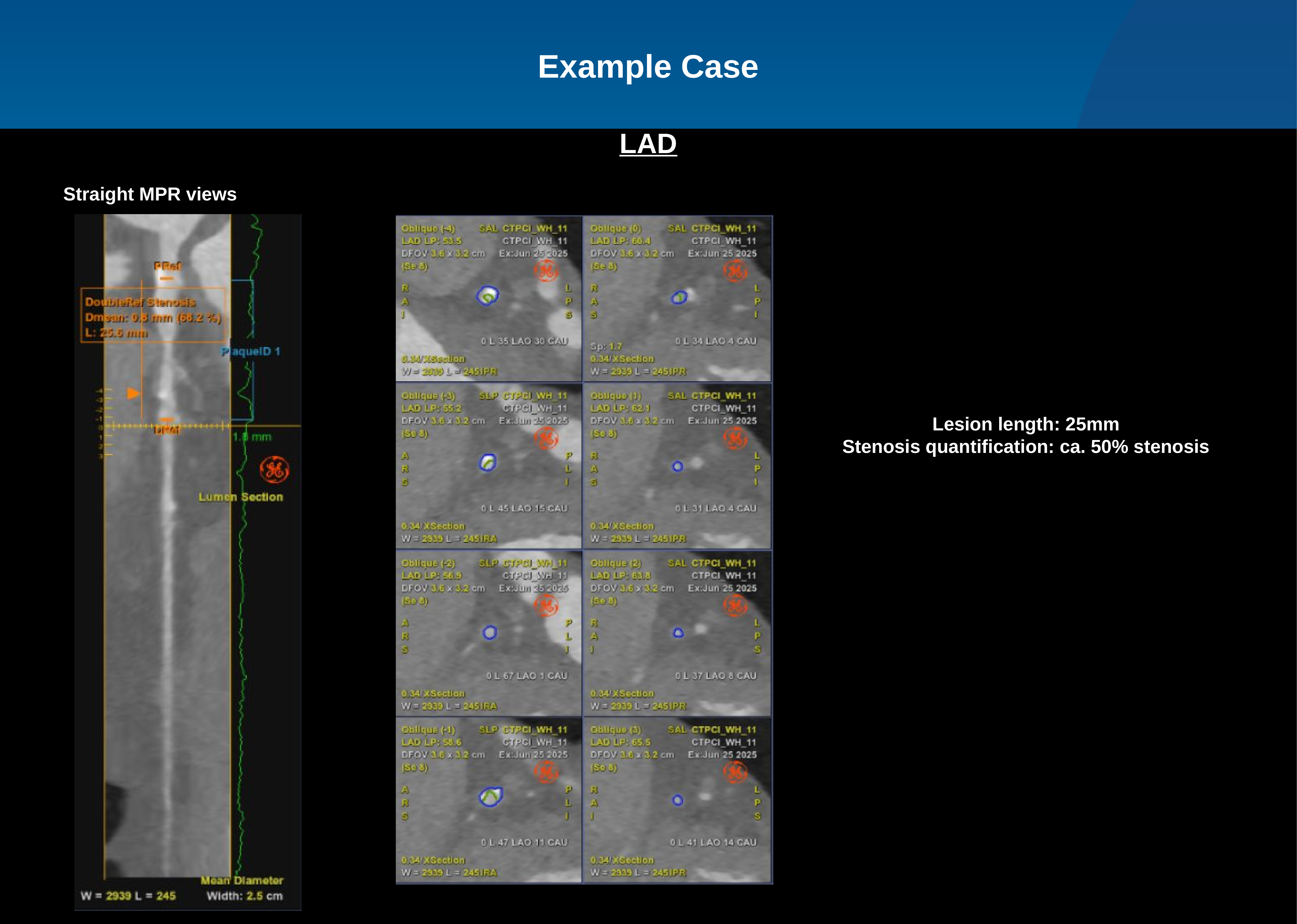

Example Case
CT-guided PCI
Case 1
LAD
Straight MPR views
Lesion length: 25mm
Stenosis quantification: ca. 50% stenosis

## Slide 7
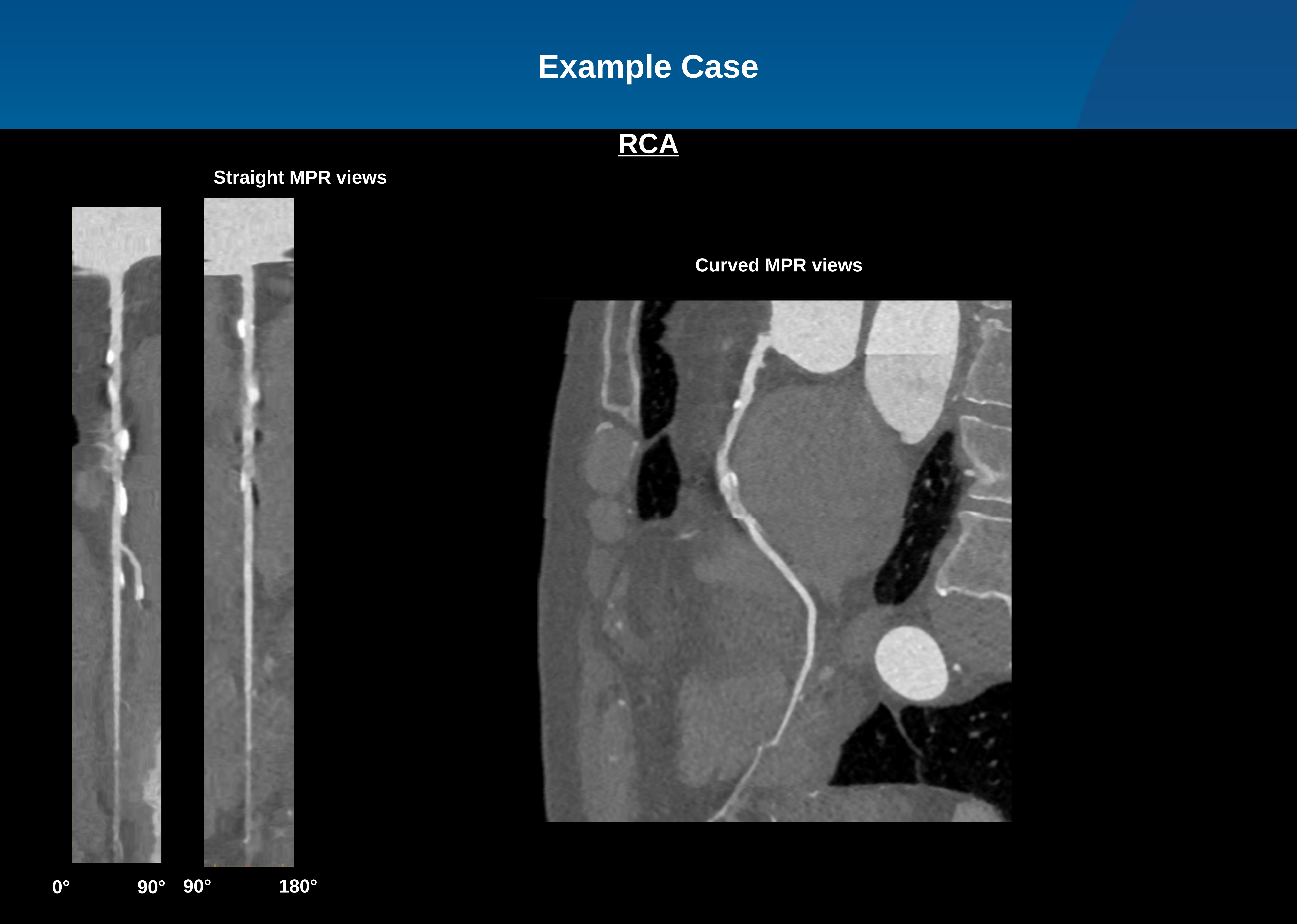

Example Case
CT-guided PCI
Case 1
RCA
Straight MPR views
Curved MPR views
90° 180°
0° 90°

## Slide 8
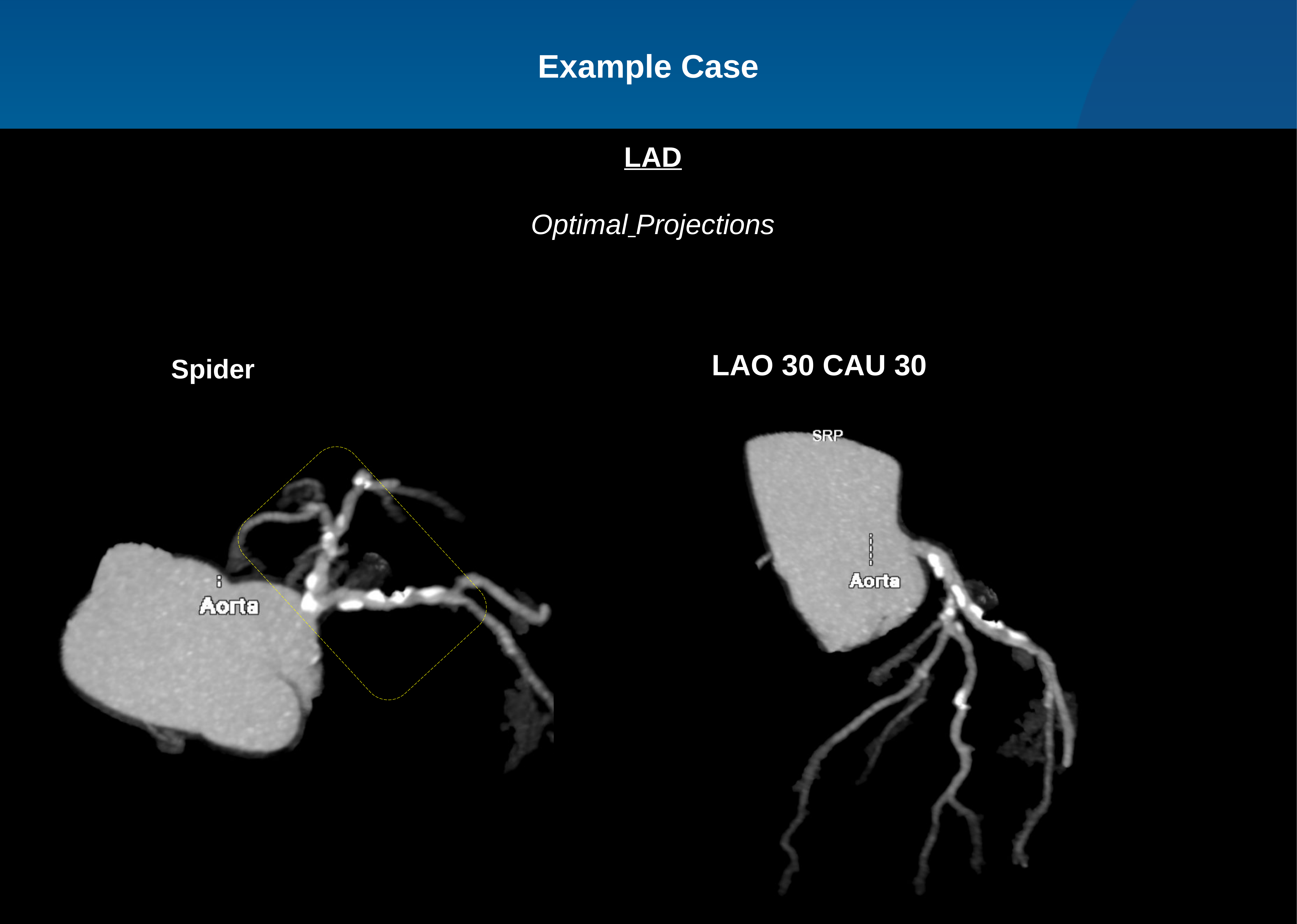

Example Case
CT-guided PCI
Case 1
LAD
Optimal Projections
LAO 30 CAU 30
Spider

## Slide 9
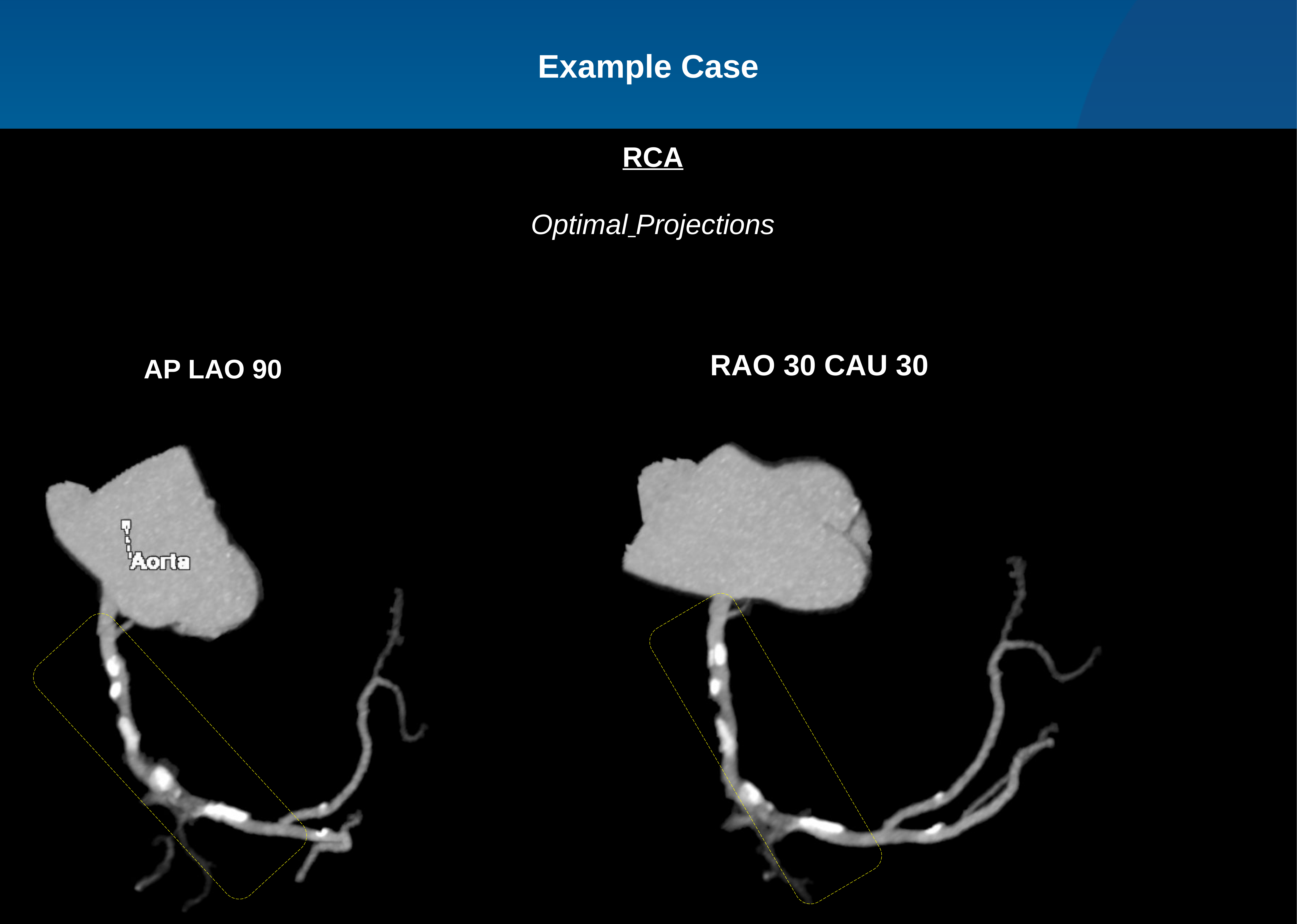

Example Case
CT-guided PCI
Case 1
RCA
Optimal Projections
RAO 30 CAU 30
AP LAO 90

## Slide 10
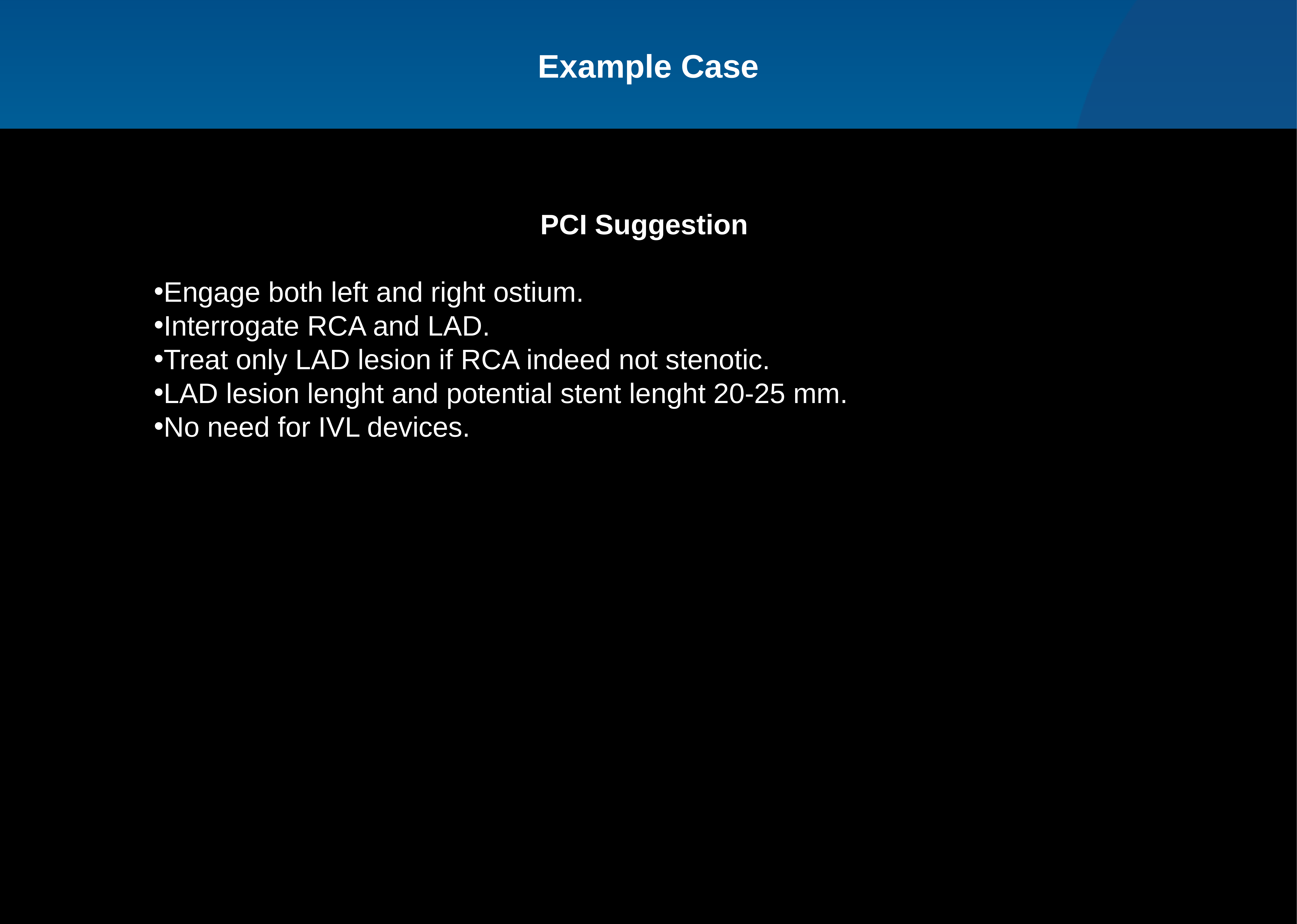

Example Case
PCI Suggestion
Engage both left and right ostium.
Interrogate RCA and LAD.
Treat only LAD lesion if RCA indeed not stenotic.
LAD lesion lenght and potential stent lenght 20-25 mm.
No need for IVL devices.
